# Object size determines the spatial spread of visual time

**DOI:** 10.1098/rspb.2016.1024

**Published:** 2016-07-27

**Authors:** Corinne Fulcher, Paul V. McGraw, Neil W. Roach, David Whitaker, James Heron

**Affiliations:** 1 Bradford School of Optometry and Vision Science, University of Bradford, BD7 1DP Bradford, UK; 2 Visual Neuroscience Group, School of Psychology, The University of Nottingham, Nottingham NG7 2RD, UK; 3 School of Optometry and Vision Sciences, University of Cardiff, Maindy Road, Cathays, Cardiff CF24 4HQ, UK

**Keywords:** time perception, spatial selectivity, duration adaptation, visual, size, after-effect

## Abstract

A key question for temporal processing research is how the nervous system extracts event duration, despite a notable lack of neural structures dedicated to duration encoding. This is in stark contrast with the orderly arrangement of neurons tasked with spatial processing. In this study, we examine the linkage between the spatial and temporal domains. We use sensory adaptation techniques to generate after-effects where perceived duration is either compressed or expanded in the opposite direction to the adapting stimulus' duration. Our results indicate that these after-effects are broadly tuned, extending over an area approximately five times the size of the stimulus. This region is directly related to the size of the adapting stimulus—the larger the adapting stimulus the greater the spatial spread of the after-effect. We construct a simple model to test predictions based on overlapping adapted versus non-adapted neuronal populations and show that our effects cannot be explained by any single, fixed-scale neural filtering. Rather, our effects are best explained by a self-scaled mechanism underpinned by duration selective neurons that also pool spatial information across earlier stages of visual processing.

## 1. Introduction

Although sub-second timing information is critical to the accuracy of most sensory and motor processing, human receptor surfaces do not appear to encode time directly in the way they initiate the analysis of non-temporal features such as pitch, location or temperature. Even at less peripheral locations within the nervous system, evidence remains sparse for any neural structures whose primary function relates to the encoding of temporal information. Despite this, we are capable of formulating temporal estimates that, although noisy [1,2] are made seemingly without conscious effort and form one of the only perceptual metrics that transcends all sensory modalities [3]. This ‘supramodal’ quality has contributed to the dominance of dedicated, modular mechanisms for time perception such as a the pacemaker-accumulator [4–6], oscillator/coincidence-detector [7,8] or memory decay [9] systems. To varying degrees, all of these systems facilitate temporal perception by monitoring ongoing background neural activity around the time of stimulus presentation.

In computational terms, centralized models have the attraction of economy in that they avoid the potentially superfluous proliferation of independent, localized timing mechanisms across primary sensory areas. However, the convergence of sensory inputs onto specialized processing modules necessitates an *a priori* pooling of information across these inputs. It therefore follows that stimulus-specific time perception of any kind presents non-trivial challenges to centralized timing processes. For sub-second duration perception, the possibility of multiple localized timing mechanisms is given credence by reports of sensory-specific distortions of perceived duration. For example, perceived visual (but not auditory) duration is compressed around the time of a saccade [10] or via repeated presentation of identical images [11]. More generally, estimates of auditory duration are expanded relative to those for visual stimuli, as well as being significantly less variable [12–15], inconsistent with a singular central mechanism for the two sensory modalities.

Further examples of sensory-specificity have been revealed by adaptation experiments where exposure to consistent duration information leads to a ‘duration after-effect’ (DAE): adaptation to relatively short/long auditory or visual durations induces perceptual expansion/compression of subsequently viewed/heard intermediate duration stimuli. These repulsion-type after-effects are bidirectional, limited to the adapting stimulus modality and tuned around the adapting duration [16–19]. The neural basis of these effects remains unclear. One possibility is that they reflect a human analogue of the ‘channel-based’ analysis predicted by neurons with bandwidth-limited duration tuning found in a range of neural structures across several amphibian and mammalian species (as recently reviewed in [20]). In the visual domain, the activity of these neurons could form a relatively late-stage, ‘dedicated’, duration-encoding mechanism [21] that—while sensory-specific—could operate at level where basic stimulus features have been pooled to allow selectivity for more complex, object-based analysis [22]. Alternatively, if visual event duration forms part of a ‘primal sketch’ [23], duration-tuned neurons would extract duration information alongside low-level stimulus features, prior to any pooling.

Here we address this question by using the orderly relationship between spatial selectivity and visual cortical hierarchy. Specifically, neurons located in extrastriate visual cortex, which perform more complex forms of visual analysis, often inherit pooled inputs from lower-level structures [24,25]. This pooling of information over larger spatial regions supports the analysis of more global image properties, produces receptive fields that are necessarily larger than their inputs and exhibit correspondingly coarser spatial selectivity. Conversely, primary sensory or (even pre-cortical) areas are more closely associated with high degrees of spatial selectivity [26–31].

By measuring the spatial tuning of DAEs, we are able to show that the effects of adaptation extend well beyond the adapted location. This broad spatial tuning could be consistent with a single, large-diameter receptive field size such as those found in the inferotemporal visual cortex [32]. However, we also show that increasing stimulus size induces a proportional increase in the width of the spatial tuning profiles. We construct a simple model based on the degree of overlap between adapted and non-adapted neural populations that allows us to quantify the scale-dependent relationship between size and adaptation spread. We propose DAEs to be a signature of mid-level visual neurons that pool spatial information across proportionally smaller lower-level inputs.

## 2. Material and methods

### (a) Observers

**Figure 1.**
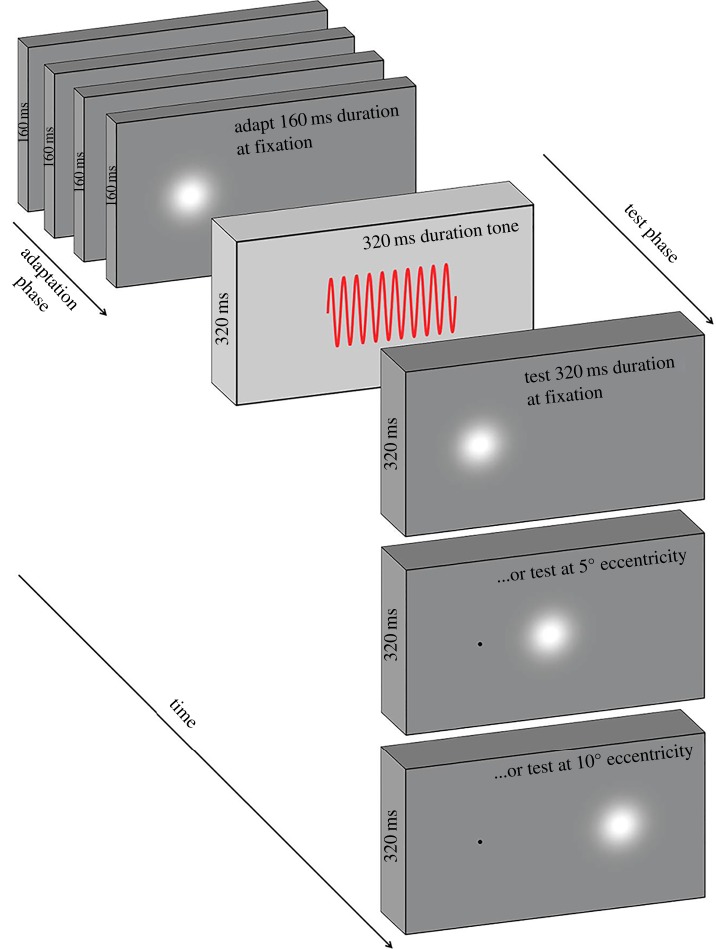
A schematic showing the adapt–test paradigm. In the adaptation phase, observers view a series of visual stimuli of fixed duration (160 ms in this example) at one of three possible adapt locations (fixation in this example). In the following test phase, observers make a duration discrimination judgement between a 320 ms auditory reference duration, and a variable visual test duration (320 ms in this example). The test stimulus may occur at fixation, at 5° eccentricity or at 10° eccentricity (constant within a block), forming nine possible adapt–test spatial configurations.

**Figure 2.**
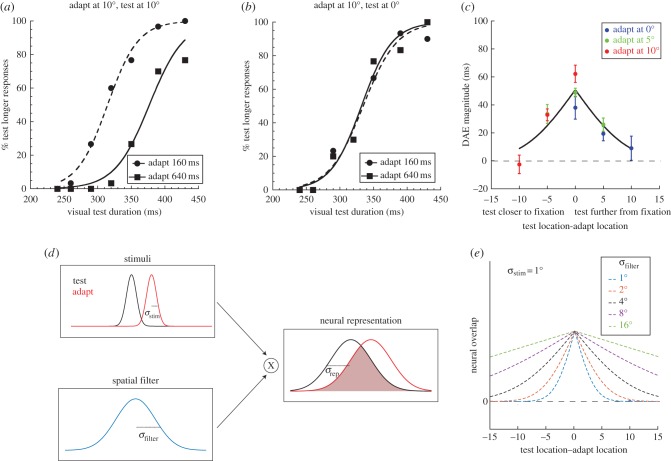
(*a*) Psychometric functions for a single representative observer making duration discrimination judgements following duration adaptation. Circles refer to the 160 ms adaptation condition and the squares show the 640 ms adaptation condition. In this condition, adapting and test duration were presented at 10° temporal to fixation. (*b*) Data from the same observer under identical conditions except for the introduction of a 10° spatial interval between adapting and testing locations. (*c*) A spatial tuning plot showing the variation in duration after-effect (DAE) magnitude across a range of adapt–test spatial configurations (see Methods and figure 2 for details). An *x*-axis value of zero represents conditions where adapt and test duration were presented at the same spatial location. Positive (negative) *x*-axis values represent conditions in which the test stimulus was presented further from (closer to) fixation than the adapting stimulus. Blue circles represent conditions where the adapting stimuli were presented at fixation, green circles represent conditions where the adapt location was 5° eccentricity and red circles represent conditions where the adapt location was 10° eccentricity. Error bars represent the SEM. (*d*), (*e*) See the main text for details.

**Figure 3.**
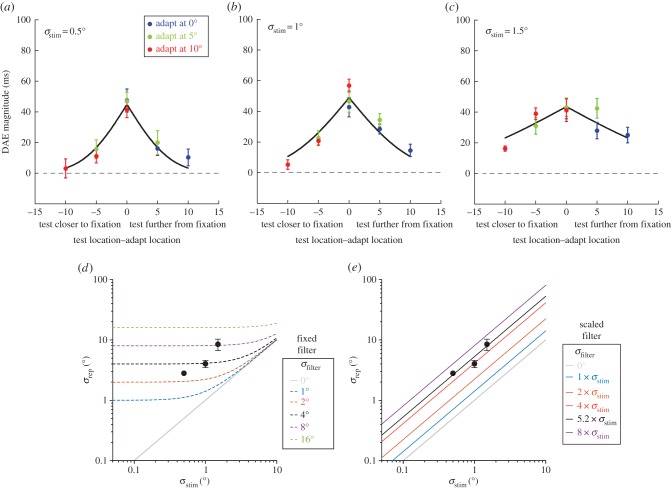
(*a–c*) Mean spatial tuning plots for the three stimulus sizes (*σ*_s_ = 0.5°, 1° and 1.5°), showing DAE magnitude as a function of the spatial separation between adapt and test locations. Blue circles represent conditions where adaption occurred at 0°, green circles represent conditions where adaptation occurred at 5° and red circles represent conditions where adaptation occurred at 10°. For each adapt–test spatial configuration, stimulus size was held constant between adapting and test phases. Error bars represent the s.e.m. (*n* = 6). (*d*), (*e*) See the main text for details.

Six observers (three naive) took part in the main experiments (figures 1–3). All observers gave their informed, written consent to participate, and had normal or corrected to normal vision and hearing at the time of the experiment.

### (b) Stimuli and apparatus

All visual stimuli were presented on a gamma-corrected Compaq P1220 CRT monitor with a refresh rate of 100 Hz and a resolution of 1280 × 1024. This was connected to a 2 × 2.26 GHz quad-core Apple Mac Pro desktop computer running Mac OS 10.6.8. All stimuli were generated using Matlab v. 7.9.0 (Mathworks, USA) running the Psychtoolbox Extension v. 3.0 (Brainard and Pelli, 1997, www.psychtoolbox.org). The physical durations of all auditory and visual stimuli were verified using a dual-channel oscilloscope. The auditory stimulus was a 500 Hz tone presented through Sennheiser HD 280 headphones. Visual stimuli were isotropic, luminance-defined Gaussian blobs (mean luminance 77 cd m^−2^) presented against a uniform grey background of 37 cd m^−2^, whose luminance (*L*) profile was defined as follows:

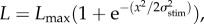
where *L*_max_ is the peak luminance value (set to 94 cd m^−2^) and *σ*_stim_ is the standard deviation of the Gaussian.

In the initial experiment (figures 2*a–c* and 3*b*) *σ*_stim_ was set to 1°. In subsequent experiments, stimulus size was modified by increasing (*σ*_stim_ = 1.5°, figure 3*c*) or decreasing (*σ*_stim_ = 0.5°, figure 3*a*) this value.

### (c) Procedure

Observers viewed the visual stimuli binocularly in a quiet, darkened room while maintaining fixation on a white 0.07° circular fixation marker presented 5.33° to the left of the centre of the screen. Viewing distance was controlled (via chin rest) to ensure one pixel subtended one arc minute. A block of trials began with an initial adapting phase consisting of 100 serially presented visual stimuli. Within a block the duration of these stimuli was fixed at either 160 ms or 640 ms. Interstimulus interval (ISI) was randomly jittered between 500 and 1000 ms. The adaptation phase was followed by a further four ‘top up’ adapting stimuli and a subsequent test phase (figure 1) consisting of a fixed (320 ms) duration auditory reference stimulus and a variable duration visual test stimulus. Observers then made a two alternative forced choice (2AFC) duration discrimination judgement as to ‘which was longer, flash or beep?’ Visual test stimuli varied in seven approximately logarithmic steps: 240, 260, 290, 320, 350, 390 and 430 ms, which were randomly interleaved within a method of constant stimuli.

Observers responded via key press, which triggered the next top-up and test cycle, until all test durations had been presented 10 times per block of trials. The adapting stimulus was presented at fixation, 5° or 10° to the right of fixation. Test stimuli were either presented at the adapting location or locations providing 5° or 10° adapt–test spatial intervals (figure 1). This provided nine adapt–test spatial configurations (three adapt locations × three test locations), each of which remained constant within a block of trials. Each adapt–test spatial configuration was repeated for both adapting durations giving a total of 18 conditions. Blocks pertaining to each condition were completed in a random order. Each observer completed three blocks per condition to give 30 repetitions per data point, per observer. In total, data collection lasted approximately 27 h per observer.

The resulting psychometric functions were fitted with a logistic function of the form

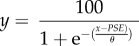
where PSE represents the point of subjective equality, corresponding to the physical test duration that is perceptually equivalent to the 320 ms auditory reference stimulus and *θ* is an estimate of the observer's duration discrimination threshold (half the difference between the values corresponding of 27 and 73% test longer responses). From these functions, PSE values were extracted for each observer for both the 160 and 640 ms adaptation conditions, across each of the nine adapt–test spatial configurations.

### (d) Modelling

To aid us in making inferences regarding the spatial scale of duration coding mechanisms, we developed a simple filtering model. We simulated the neural representation (rep) of each stimulus across retinotopic cortex by convolving its horizontal contrast envelope with a Gaussian spatial filter

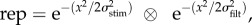
where *σ*_stim_ and *σ*_filt_ are the standard deviations of the stimulus and filter, respectively, and *x* indicates the spatial distance from the centre of the stimulus/filter (all in degrees of visual angle).

Because both stimulus and filter are Gaussians, rep is itself a Gaussian centred at the location of the stimulus, with a standard deviation *σ*_rep_ given by

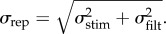


The proportional overlap *O* between adapting and test neural representations can be calculated by

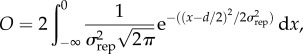
where *d* is the centre-to-centre distance between adapting and test stimuli.

The expected DAE was assumed to be a linear function of this overlap

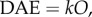
where *k* is the peak DAE obtained with identical adapting and test stimuli.

For each stimulus size, we fitted the spatial filter model to the tuning function relating DAE magnitude to separation, finding the values of *σ*_rep_ and *k* that minimized the sum of squared residual errors between expected and measured after-effect magnitudes.

## 3. Results

Figure 2*a* shows sample psychometric functions from a single representative observer. The proportion of responses where the visual test was perceived as longer than the auditory reference is plotted as a function of visual test duration for the condition where both the adapting stimulus and test stimuli were presented at 10° from fixation (i.e. with no spatial separation). Repeated presentations of the 640 ms adapting stimulus (solid black curve, black squares) depresses the number of ‘test longer than reference’ responses, which reflects a perceived compression in the duration of the test stimulus: a physical test duration of 377 ms is judged as perceptually equivalent to a physical auditory reference duration of 320 ms. Conversely, the function relating to the 160 ms adaptation condition (dashed curve, black circles) is shifted leftwards, reflecting an expansion of the perceived duration of the test stimulus: a physical test stimulus of 315 ms now has perceptual equivalence with the reference stimulus. These temporal distortions are consistent with previous reports of bi-directional, repulsive DAEs [17,19].

The extent of the lateral separation between the two functions provides a measure of DAE magnitude and can be expressed as the arithmetic difference between PSE values for the two adapting duration conditions

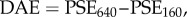
where PSE_640_ is the PSE value obtained from the 640 ms adapting duration and PSE_160_ is the PSE value obtained from the 160 ms adapting duration. For the observer shown in figure 2*a*, DAE = 62 ms when adapting and test durations are both presented at the same location. Of particular interest in this study was to establish how DAE varied during manipulation of the adapt–test spatial interval. Figure 2*b* shows psychometric functions for the same observer when the adapting and test stimuli were separated by 10° (‘Adapt at 10°, test at fixation’). The superimposition of the two functions is in stark contrast with the lateral separation shown in figure 2*a*. This represents a reduction in the effectivity of the adapting stimuli: the perceived duration of the test stimulus shows negligible variation across both adapting durations.

Figure 2*c* shows data from the same observer where DAE is plotted as a function of all nine adapt–test spatial configurations. For all three adapting locations, robust DAEs are generated by presenting adapt and test stimuli at the same spatial location (figure 2*c*, central data points). As the adapt–test spatial interval is increased, DAE magnitude shows a progressive decrease, indicating a reduction in the perceptual bias induced by adaptation. This pattern of spatial tuning is manifest for all three adapting locations, as demonstrated by the red, green and blue data points forming a single function.

Spatially tuned DAEs are evidence that—at some level—event timing must be segregated into distinct regions of visual space, a finding that could signal the presence of neurons that are selective for both the duration *and* spatial location of a visual event. But what is the spatial scale of duration coding mechanisms? To address this question quantitatively, we developed a simple spatial filtering model based on the assumption that DAEs occur when (and only when) adapting and test stimuli stimulate overlapping neural populations (see Material and methods for details). As illustrated in figure 2*d*, we first convolved the horizontal contrast profiles of our stimuli with a Gaussian filter corresponding to neural blur, then calculated the proportional overlap between the resulting neural representations of the adapt and test stimuli. The proportion of overlap was then calculated for a range of different adapt–test spatial separations. Figure 2*e* shows the resulting spatial tuning functions obtained with a range of neural representation sizes. Application of the model to the individual data shown in figure 2*c*, revealed a best-fitting *σ*_rep_ of 3.67°, which is several multiples of *σ*_stim_ (the spatial spread of the stimulus). In other words, duration adaptation extends into spatial regions well beyond the physical confines of the adapting stimuli themselves.

A relatively large after-effect spread across space could be consistent with late-stage processing subserved by a coarse, fixed scale of spatial filtering [33]. If this scale (*σ*_filter_) is larger than the stimulus, (*σ*_stim_—as depicted in figure 2*d*) the degree of overlap between adapting and test neural representations (*σ*_rep_) would be similar across modest changes in stimulus sizes above and below 1°. We examined this possibility by repeating our experiment using smaller (0.5°) and larger (1.5°) Gaussian stimuli. Group averaged results for each of the three size conditions are shown in figure 3*a–c*. Irrespective of stimulus size, DAE magnitude declines systematically with adapt–test spatial interval; however, the rate of decline varies with stimulus size. This progressive broadening of spatial tuning with increasing stimulus size is summarized in figure 3*d*, where best-fitting *σ*_rep_ values are plotted as a function of *σ*_stim_. In comparison, the dotted lines show a family of model predictions for different levels of neural blur. Clearly, changes in the spatial tuning of the DAE with stimulus size are not consistent with any fixed scale of spatial filtering.

From the best-fitting *σ*_rep_ values, we can work back in our model to calculate the neural blur of the filter *σ*_filter_, which would have produced this pattern of results. The data predict filter sizes of 2.76°, 3.91° and 7.86° for our three stimulus sizes of 0.5°, 1° and 1.5°. Rather than a fixed level of coarse spatial filtering, this suggests a ‘self-scaled’ relationship in which the spatial scale of the filter determining after-effect tuning forms a multiple of the spatial scale of the stimulus. Simulations based on this principle are shown in figure 3*e* where the best-fitting scaled filter is 5.2 × *σ*_stim_ (figure 3*e*—black line).

## 4. Discussion

We sought to investigate the interaction between spatial information, recent sensory history and the perception of duration. Adaptation techniques were used to generate bidirectional repulsive DAEs, which were tested for their sensitivity to adapt–test changes in spatial location. This sensitivity was found to be coarse: the effects of adaptation spread into a region considerably larger than the adapting stimulus itself (figures 2*c* and 3*b*). The size of this region is proportional to the size of the adapting stimulus (figure 3*a–c*). Our model simulations allowed us to assess our spatial tuning data alongside predictions based on a range of fixed, coarse-scale spatial filters (figure 3*d*) versus scaled filtering which forms a multiple of stimulus size (figure 3*e*). Fixed-scale filters were unable to capture the relationship between stimulus size and after-effect spread. Instead, our data are better described by modelling based on the principle that DAEs are generated by a mechanism with self-scaled filtering properties. The effect of this self-scaling is to spread DAEs across an area that is approximately five-times larger than the adapting stimulus.

Broad spatial tuning has practical implications for how adaptation-induced biases are measured. Because duration adaptation do not transfer between sensory modalities [17], our observers judged the perceived duration of a visual test stimulus relative to an auditory reference. An alternative is to use a visual reference that is presented at an unadapted spatial location. However, our data show that it is critical to sufficiently separate the stimuli (particularly if the stimuli themselves are large), otherwise adaptation will influence both the reference and test stimuli during the 2AFC judgement. This provides a possible explanation for why robust DAEs have not been reported in experiments using large visual test and reference stimuli presented in relatively close spatial proximity [34].

The spatial tuning reported here contradicts the conclusions of a very recent study where after-effects were generated in one hemisphere (e.g. 10° left of fixation) and then tested in the opposite hemisphere (e.g. 10° right of fixation) [35]. In the Li *et al.* study, adapting and test stimuli were always presented at 10° either side of fixation. This raises the possibility that interhemispheric communication between corresponding areas of cortical eccentricity (e.g. [36]) could facilitate the transfer of DAEs around an iso-eccentric annulus centred on fixation. This scenario would produce spatial tuning across the annulus' diameter (as per this study) but not around its circumference (as per the Li *et al.* study). To investigate this possibility, we repeated our experiment using a 0.5° sized stimulus and a 20° adapt–test spatial interval that spanned 10° either side of fixation. The results are shown in the electronic supplementary material, figure S1. In keeping with earlier experiments, (figure 3*a–c*) all observers show robust DAEs when adapting and test stimuli were both presented 10° right of fixation. However, no significant after-effects were generated when adapting stimuli were presented at 10° right of fixation and test stimuli were presented 10° left of fixation, despite matching eccentricity across hemispheres. This is consistent with a spatial filtering account of our ‘within-hemisphere’ data (figure 3*a*), which predicts a negligible (more than 5%) after-effect magnitude for the 0.5° sized stimulus across a 20° adapt–test spatial interval.

At the opposite extreme to position-invariant accounts of temporal processing, effects are generated when observers view continuous periods of temporally dynamic (flickering or drifting) visual patterns. Subsequently viewed test stimuli typically undergo perceptual *compression,* (but see [37]) within the same region of the visual field [38,39]. These after-effects show very narrow (approx. 1°) spatial tuning [40] and no interocular transfer, leading some to propose an adaptation locus within the magnocellular layers of the LGN ([41], but see [42]). Similarly ‘repetition suppression’ paradigms show that the presentation of two or more identical visual stimuli in close temporal proximity leads the underestimation of the second stimulus' duration [43]. This effect is exaggerated when the two stimuli share the same orientation and are presented within approximately 2° of each another. Again, these effects have been attributed to mechanisms driven by early striate visual neurons [44].

This group of duration phenomena appear to share some common features: unidirectional (mostly compressive) perceptual distortion, which is tightly tuned to low-level stimulus characteristics such as spatial location. These features contrast sharply with the DAEs reported here which could suggest that the two types of after-effect (unidirectional, narrowly tuned versus bidirectional, broadly tuned) might be signatures of distinct temporal processing mechanisms.

However, recent advances in our understanding of visual spatial adaptation offer an alternative interpretation. Adaptation to stimulus features such as contrast, temporal frequency, motion and orientation modulates neural activity across a wide range of areas from the retina, to the striate and extrastriate cortices (as recently reviewed in [45]). Neurophysiological advances have revealed an adaptation cascade where the activity at any given site is a product of adaptation intrinsic to neurons at that site *and* adaptation inherited from earlier visual areas [46,47]. In some cases [47,48], the ‘downstream’ recipients of ‘upstream’ adaptation are unable to distinguish between adapted and non-adapted inputs, leading to a cumulative superimposition of distinct adaptation effects [49,50].

Could adaptation effects from different levels of neural processing also occur for temporal information? Because receptive field size increases systematically throughout pre-cortical, striate and extrastriate visual areas [26–30], our broad spatial tuning dictates that bidirectional, repulsive DAEs must originate at a cortical location beyond that responsible for the narrowly tuned, unidirectional effects discussed above. Whatever the relationship between these two after-effects, simple inheritance of earlier adaptation would predict that our repulsive DAEs should display similarly narrow spatial tuning [24,51]. Instead, our tuning profiles suggest repulsive DAEs are generated by subsequent phase of adaptation that is embodied with the spatial selectivity of neurons whose larger receptive field size reflects their downstream location [46,52,53]. In this context, the output duration signal from early mechanisms [39,43,44] would feed forward to form the (compressed) input signal for a downstream mechanism responsible for the repulsion-type after-effects reported here.

As argued elsewhere [17], channel-based duration encoding by neurons with bandwidth-limited sensitivity to a range of durations [54] is consistent with repulsion-type after-effects. In the visual domain, a relevant example is the duration tuning seen across the millisecond range in ‘off response’ neurons within areas 17 and 18 of cat visual cortex [55]. Within these regions (and their primate homologues V1 and V2), individual neurons show tuning for a raft of stimulus features such as orientation, spatial frequency, contrast and motion [56,57]. Neurons with bandpass duration selectivity have also been documented in the auditory systems of a wide range of species including cat auditory cortex [58], the auditory midbrain nuclei of amphibians [59], bats [60,61], guinea pigs [62,63], rats [64] and mice [65]. In addition to stimulus duration, these same neurons invariably show selectivity for auditory pitch [20] and, in some cases, spatial location [66]. Cross-species and cross-sensory modality generality points towards duration being a generic feature to which a wide variety of neurons can show tuning.

Which neurons might be responsible for mediating channel-based processing of duration in humans? Recent neurophysiological evidence suggests a duration processing role for sub-regions within the inferior parietal lobule [67–69]. However, visually responsive parietal areas have large, often bilateral receptive fields [70], the vast majority of which are at least 5° in diameter [71–73]. It therefore seems likely that the adaptation-induced perceptual distortions described here and elsewhere [37,39,43,44] reflect intrinsic adaptation in upstream visual areas, which undergo subsequent duration encoding in extrastriate areas such as LIP and SMG. Motor, premotor and supplementary motor cortices are also reported to show duration-dependent patterns of neural activity [74–76] but again, how intrinsic duration adaptation within these areas could facilitate even broadly tuned spatial specificity (or indeed perceptual distortions in the absence of any motor action) remains unclear.

When considering the neural underpinnings of DAEs, it is important to acknowledge the relationship between stimulus size and spatial tuning (figure 3). This size dependency is incompatible with the uniformly broad tuning predicted by a large fixed-scale spatial filter that encodes duration across a range of stimulus sizes (see horizontal sections of dashed lines in figure 3*d*). Is there any evidence for a visual processing stage which not only summates low-level information across a moderate spatial extent, but also whose scale is fundamentally linked to the scale of its inputs? A prime example of exactly this relationship is provided by the interdependency between mechanisms encoding spatial variations in luminance (first-order) and those encoding variations in texture/contrast (second-order). It is widely accepted that the rectified output of small, linear first-order filters form the input to subsequent, larger second-order filters (for a recent review see [77]). To extract contrast/texture modulations each second-order filter performs ‘spatial pooling’ by combining the outputs of several neighbouring first-order filters [78,79]. As a result, second-order perceptual phenomena are more spatially diffuse than their first-order counterparts [80–82].

Critically, second-order pooling of first-order inputs creates spatial scale-dependency between the two stages: second-order filter size forms a multiple of its first-order input [83]. Psychophysical estimates place this multiple between 3 and 50 [82,84–86], dependent on the stimulus and task [87]. Single-unit recordings have demonstrated that this relationship is underpinned by neurons whose spatial frequency tuning for contrast or texture-defined information is between 5 and 30 × lower than for luminance-defined information [88–90].

If DAEs are indeed a product of duration tuning within neurons also selective for second-order image statistics then two clear predictions follow: (i) after-effects should propagate into a region larger than that predicted by first-order filtering (i.e. the borders of the stimulus itself) and (ii) the size of this region will be a fixed multiple of adapting stimulus size, reflecting the proportionality between first- and second-order size tuning. Our data and model simulations show precisely this effect. Ongoing experiments in our laboratory will test a further prediction of the second-order hypothesis: it should be possible to induce DAEs by adapting to repeated presentations of fixed-duration second-order information (e.g. sinusoidal contrast modulation) superimposed on first-order information which does not provide any consistent duration signal (e.g. dynamic luminance noise). In this situation, the adapting duration signal would be available to second-order mechanisms alone and its effects would therefore only be manifest with second-order test stimuli. This scenario would be compatible with a recent report of DAEs transferring across first-order orientation [91].

In summary, our data and model are suggestive of a mid-level form of duration encoding by visual neurons that are selective for a stimulus' spatial characteristics and its duration. These behavioural data are consistent with neurophysiological evidence of neurons showing bandwidth-limited tuning to duration alongside a raft of other stimulus features across a wide range of species. Although such a mechanism has the apparent disadvantage of relatively coarse spatial resolution, it could provide duration estimates that avoid some of the ambiguities associated with the earliest stages of visual processing. For example, using first-order luminance alone during object identification can yield spurious results that are corrupted by shadows and shading gradients [92]. By pooling across a larger spatial area, it is possible to disambiguate object–background borders via second-order changes in texture or contrast. Relatedly, changes in viewing distance alter absolute first- and second-order spatial scale but, for any given object, the size ratio between these cues does not change. This ‘scale invariance’ [93–95] ensures that our ability to detect and discriminate between stimulus features defined by second-order cues remains constant across distances in a way that does not hold for first-order cues [96]. Therefore, if duration selectivity *were* a feature of neurons tasked with more complex image attributes it would afford perceived duration a degree of object specificity that could be robust enough to cope with occasions where lower-level information is less reliable. Studies examining after-effects of temporal perception while systematically varying stimulus feature complexity will help localize the strata occupied by time perception within the sensory processing hierarchy.

## Supplementary Material

Supp Fig 1 cross hemisphere.jpg

## Supplementary Material

Cross hemifield control experiment

## Data accessibility

The datasets supporting this article have been uploaded as part of the electronic supplementary material.
