# Supplementary figures and images for: Object size determines the spatial spread of visual time
Source: Proc Biol Sci. 2016 Jul 27;283(1835):20161024. doi: 10.1098/rspb.2016.1024 (PMC4971211; doi:10.1098/rspb.2016.1024)

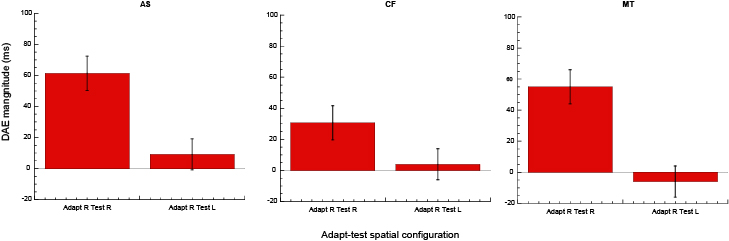

Supplement: Supp Fig 1 cross hemisphere.jpg [file rspb20161024supp1.jpg]
